# Supplementary material for: Comparing Virtual and Center-Based Cardiac Rehabilitation on Changes in Frailty
Source: Int J Environ Res Public Health. 2023 Jan 14;20(2):1554. doi: 10.3390/ijerph20021554 (PMC9865753; doi:10.3390/ijerph20021554)
Supplement: Supplementary file 1 [file ijerph-20-01554-s001.zip › ijerph-2147161-supplementary.pdf]

# Supplemental Tables Package

**Supplemental Table S1:** Description of center- and virtual-based CR[a] programs[b]

| CR[a] Model | Center-based CR[a] | Virtual CR[a] |
|---|---|---|
| *Duration of program* | 6-weeks | 9-10 weeks |
| *Exercise component* | Group-based exercise sessions which included individualized prescription based on graded exercise stress testing at CR admission and orthopaedic limitations. | Individual home-based exercise prescribed using graded exercise stress test results at CR admission, orthopaedic limitations, and availability of exercise equipment/resources to the patient. |
| *Type of exercise* | Exercises include a continuous or interval type aerobic exercise on a treadmill, leg cycle ergometer, or arm cycle ergometer. | CR physiotherapists prescribe exercise types by discussing the equipment or resources that are available on a patient-to-patient basis. Examples of exercises include walking indoors or outdoors, bicycling, recreational sport, body weight resistance training, resistance training with equipment, and flexibility or stretching exercises. |
| *Frequency & duration* | Once weekly, 60-minute exercise class, including 10 minutes of warm-up, 40 minutes of exercise time, and 10 minutes of cool-down. Participants were encouraged to reach 150 minutes of moderate-vigorous exercise per week by supplementing outside of CR. | Exercise target to meet 150 minutes of moderate-vigorous exercise per week. This exercise target could be completed in bouts of 10+ minutes of exercise throughout the week. |
| *Education component* | Up to 3, one-hour, weekly group-based phone/video call rotation from team physiotherapist, nurse, and dietitian. Education sessions focussed on cardiovascular health and risk factor reduction, incorporating health behaviour changes to diet, physical activity, or medications if needed. In addition to weekly phone calls, center-based participants could interact with members of their CR team during each of their 6 exercise sessions, totalling a possible 9 hours spent with CR staff. | Up to 4, one-hour, weekly group-based phone/video call rotation from team physiotherapist, nurse, and dietitian, with a group Question and Answer Zoom video session held during week 4. From week 5 to program completion, virtual participants received up to 6 weekly, 45-minute, individual phone calls, totalling a possible 8.5 hours spent with CR staff. Sessions provided education on cardiovascular health and risk factor reduction, incorporating health behaviour changes to diet, physical activity, or medications if needed. |
| *Multidisciplinary healthcare team* | Medical director, program lead, nurse, physiotherapist, dietitian. | Medical director, program lead, nurse, physiotherapist, dietitian. |

[a]Abbreviations: CR, cardiac rehabilitation. [b]Table descriptions are from the Nova Scotia Health Hearts and Health in Motion CR Program.

**Supplemental Table S2:** Frequency of 65 CLSA-FI items for CR participants at admission & follow-up

| **Question** | **Deficit** | **Coding** | **Frequency N (sample %)** | | | |
|---|---|---|---|---|---|---|
| | | | **Center-based CR Responses** | | **Virtual CR Responses** | |
| | | | Admission | Follow-up | Admission | Follow-up |
| 1) Can you.. | Dress and undress yourself? | 0 = Yes, without help.<br>0.5 = Yes, with some help.<br>1 = No, unable to do so. | 0 = 41 (97.6%)<br>0.5 = 1 (2.4%)<br>1 = 0 (0.0%) | 0 = 41 (97.6%)<br>0.5 = 1 (2.4%)<br>1 = 0 (0.0%) | 0 = 25 (100.0%)<br>0.5 = 0 (0.0%)<br>1 = 0 (0.0%) | 0 = 25 (100.0%)<br>0.5 = 0 (0.0%)<br>1 = 0 (0.0%) |
| | Take care of your own appearance? | 0 = Yes, without help.<br>0.5 = Yes, with some help.<br>1 = No, unable to do so. | 0 = 42 (100.0%)<br>0.5 = 0 (0.0%)<br>1 = 0 (0.0%) | 0 = 42 (100.0%)<br>0.5 = 0 (0.0%)<br>1 = 0 (0.0%) | 0 = 24 (96.0%)<br>0.5 = 1 (4.0%)<br>1 = 0 (0.0%) | 0 = 25 (100.0%)<br>0.5 = 0 (0.0%)<br>1 = 0 (0.0%) |
| | Walk? | 0 = Yes, without help.<br>0.5 = Yes, with some help.<br>1 = No, unable to do so. | 0 = 41 (97.6%)<br>0.5 = 1 (2.4%)<br>1 = 0 (0.0%) | 0 = 41 (97.6%)<br>0.5 = 1 (2.4%)<br>1 = 0 (0.0%) | 0 = 25 (100.0%)<br>0.5 = 0 (0.0%)<br>1 = 0 (0.0%) | 0 = 25 (100.0%)<br>0.5 = 0 (0.0%)<br>1 = 0 (0.0%) |
| | Get in and out of bed? | 0 = Yes, without help.<br>0.5 = Yes, with some help.<br>1 = No, unable to do so. | 0 = 42 (100.0%)<br>0.5 = 0 (0.0%)<br>1 = 0 (0.0%) | 0 = 42 (100.0%)<br>0.5 = 0 (0.0%)<br>1 = 0 (0.0%) | 0 = 25 (100.0%)<br>0.5 = 0 (0.0%)<br>1 = 0 (0.0%) | 0 = 25 (100.0%)<br>0.5 = 0 (0.0%)<br>1 = 0 (0.0%) |
| | Take a bath or shower | 0 = Yes, without help.<br>0.5 = Yes, with some help.<br>1 = No, unable to do so. | 0 = 42 (100.0%)<br>0.5 = 0 (0.0%)<br>1 = 0 (0.0%) | 0 = 41 (97.6%)<br>0.5 = 1 (2.4%)<br>1 = 0 (0.0%) | 0 = 24 (96.0%)<br>0.5 = 1 (4.0%)<br>1 = 0 (0.0%) | 0 = 25 (100.0%)<br>0.5 = 0 (0.0%)<br>1 = 0 (0.0%) |
| 2) Can you.. | Use the telephone? | 0 = Yes, without help.<br>0.5 = Yes, with some help.<br>1 = No, unable to do so. | 0 = 42 (100.0%)<br>0.5 = 0 (0.0%)<br>1 = 0 (0.0%) | 0 = 42 (100.0%)<br>0.5 = 0 (0.0%)<br>1 = 0 (0.0%) | 0 = 25 (100.0%)<br>0.5 = 0 (0.0%)<br>1 = 0 (0.0%) | 0 = 25 (100.0%)<br>0.5 = 0 (0.0%)<br>1 = 0 (0.0%) |
| | Get places out of walking distance? | 0 = Yes, without help.<br>0.5 = Yes, with some help.<br>1 = No, unable to do so. | 0 = 41 (97.6%)<br>0.5 = 1 (2.4%)<br>1 = 0 (0.0%) | 0 = 41 (97.6%)<br>0.5 = 1 (2.4%)<br>1 = 0 (0.0%) | 0 = 24 (96.0%)<br>0.5 = 1 (4.0%)<br>1 = 0 (0.0%) | 0 = 24 (96.0%)<br>0.5 = 1 (4.0%)<br>1 = 0 (0.0%) |

| Question | Deficit | Coding | Frequency N (sample %) | | | |
|---|---|---|---|---|---|---|
| | | | Center-based CR Responses | | Virtual CR Responses | |
| | | | Admission | Follow-up | Admission | Follow-up |
| | Go shopping for groceries and clothes? | 0 = Yes, without help.<br>0.5 = Yes, with some help.<br>1 = No, unable to do so. | 0 = 39 (7.1%)<br>0.5 = 3 (92.9%)<br>1 = 0 (0.0%) | 0 = 41 (97.6%)<br>0.5 = 1 (2.4%)<br>1 = 0 (0.0%) | 0 = 23 (92.0%)<br>0.5 = 2 (8.0%)<br>1 = 0 (0.0%) | 0 = 23 (92.0%)<br>0.5 = 2 (8.0%)<br>1 = 0 (0.0%) |
| | Prepare your own meals? | 0 = Yes, without help.<br>0.5 = Yes, with some help.<br>1 = No, unable to do so. | 0 = 42 (100.0%)<br>0.5 = 0 (0.0%)<br>1 = 0 (0.0%) | 0 = 42 (100.0%)<br>0.5 = 0 (0.0%)<br>1 = 0 (0.0%) | 0 = 24 (96.0%)<br>0.5 = 1 (4.0%)<br>1 = 0 (0.0%) | 0 = 23 (92.0%)<br>0.5 = 2 (8.0%)<br>1 = 0 (0.0%) |
| | Do your housework? | 0 = Yes, without help.<br>0.5 = Yes, with some help.<br>1 = No, unable to do so. | 0 = 38 (90.5%)<br>0.5 = 4 (9.5%)<br>1 = 0 (0.0%) | 0 = 39 (7.1%)<br>0.5 = 3 (92.9%)<br>1 = 0 (0.0%) | 0 = 24 (96.0%)<br>0.5 = 1 (4.0%)<br>1 = 0 (0.0%) | 0 = 23 (92.0%)<br>0.5 = 2 (8.0%)<br>1 = 0 (0.0%) |
| | Take your own medicine? | 0 = Yes, without help.<br>0.5 = Yes, with some help.<br>1 = No, unable to do so. | 0 = 40 (95.2%)<br>0.5 = 2 (4.8%)<br>1 = 0 (0.0%) | 0 = 42 (100.0%)<br>0.5 = 0 (0.0%)<br>1 = 0 (0.0%) | 0 = 25 (100.0%)<br>0.5 = 0 (0.0%)<br>1 = 0 (0.0%) | 0 = 25 (100.0%)<br>0.5 = 0 (0.0%)<br>1 = 0 (0.0%) |
| | Handle your own money? | 0 = Yes, without help.<br>0.5 = Yes, with some help.<br>1 = No, unable to do so. | 0 = 40 (95.2%)<br>0.5 = 2 (4.8%)<br>1 = 0 (0.0%) | 0 = 40 (95.2%)<br>0.5 = 2 (4.8%)<br>1 = 0 (0.0%) | 0 = 24 (96.0%)<br>0.5 = 1 (4.0%)<br>1 = 0 (0.0%) | 0 = 23 (92.0%)<br>0.5 = 2 (8.0%)<br>1 = 0 (0.0%) |
| 3) Do you have difficulty with.. | Reaching or extending your arms above your shoulders? | 0 = No.<br>0.25 = Yes, a little difficult.<br>0.5 = Yes, somewhat difficult.<br>0.75 = Yes, very difficult.<br>1 = Unable to do so.<br>1 = Do not do on doctor's orders. | 0 = 32 (76.2%)<br>0.25 = 5 (11.9%)<br>0.5 = 4 (9.5%)<br>0.75 = 0 (0.0%)<br>1 = 1 (2.4%) | 0 = 34 (81%)<br>0.25 = 3 (7.1%)<br>0.5 = 3 (7.1%)<br>0.75 = 1 (2.4%)<br>1 = 1 (2.4%) | 0 = 14 (56.0%)<br>0.25 = 9 (36.0%)<br>0.5 = 1 (4.0%)<br>0.75 = 0 (0.0%)<br>1 = 1 (4.0%) | 0 = 16 (64.0%)<br>0.25 = 6 (24.0%)<br>0.5 = 2 (8.0%)<br>0.75 = 0 (0.0%)<br>1 = 1 (4.0%) |

| Question | Deficit | Coding | Frequency N (sample %) | | | |
|---|---|---|---|---|---|---|
| | | | Center-based CR Responses | | Virtual CR Responses | |
| | | | Admission | Follow-up | Admission | Follow-up |
| | Stooping, crouching, or kneeling down? | 0 = No.<br>0.25 = Yes, a little difficult.<br>0.5 = Yes, somewhat difficult.<br>0.75 = Yes, very difficult.<br>1 = Unable to do so.<br>1 = Do not do on doctor's orders. | 0 = 28 (66.7%)<br>0.25 = 6 (14.3%)<br>0.5 = 4 (9.5%)<br>0.75 = 4 (9.5%)<br>1 = 0 (0.0%) | 0 = 27 (64.3%)<br>0.25 = 7 (16.7%)<br>0.5 = 6 (14.3%)<br>0.75 = 2 (4.8%)<br>1 = 0 (0.0%) | 0 = 11 (44.0%)<br>0.25 = 8 (32.0%)<br>0.5 = 3 (12.0%)<br>0.75 = 3 (12.0%)<br>1 = 1 (4.0%) | 0 = 13 (52.0%)<br>0.25 = 5 (20.0%)<br>0.5 = 2 (8.0%)<br>0.75 = 4 (16.0%)<br>1 = 1 (4.0%) |
| | Pushing or pulling large objects like a living room chair? | 0 = No.<br>0.25 = Yes, a little difficult.<br>0.5 = Yes, somewhat difficult.<br>0.75 = Yes, very difficult.<br>1 = Unable to do so.<br>1 = Do not do on doctor's orders. | 0 = 29 (76.2%)<br>0.25 = 8 (19.0%)<br>0.5 = 2 (4.8%)<br>0.75 = 3 (7.1%)<br>1 = 0 (0.0%) | 0 = 37 (88.1%)<br>0.25 = 0 (0.0%)<br>0.5 = 0 (0.0%)<br>0.75 = 0 (0.0%)<br>1 = 4 (9.5%)<br>Missing = 1 (2.4%) | 0 = 16 (64.0%)<br>0.25 = 7 (28.0%)<br>0.5 = 2 (8.0%)<br>0.75 = 0 (0.0%)<br>1 = 0 (0.0%) | 0 = 18 (72.0%)<br>0.25 = 5 (20.0%)<br>0.5 = 2 (8.0%)<br>0.75 = 0 (0.0%)<br>1 = 0 (0.0%) |
| | Lifting 10 lbs. ( or 4.5kg) from the floor, like a heavy bag of groceries? | 0 = No.<br>0.25 = Yes, a little difficult.<br>0.5 = Yes, somewhat difficult.<br>0.75 = Yes, very difficult.<br>1 = Unable to do so.<br>1 = Do not do on doctor's orders. | 0 = 33 (78.6%)<br>0.25 = 4 (9.5%)<br>0.5 = 2 (4.8%)<br>0.75 = 2 (4.8%)<br>1 = 1 (2.4%) | 0 = 36 (85.7%)<br>0.25 = 4 (9.5%)<br>0.5 = 0 (0.0%)<br>0.75 = 2 (4.8%)<br>1 = 0 (0.0%) | 0 = 23 (92.0%)<br>0.25 = 2 (8.0%)<br>0.5 = 0 (0.0%)<br>0.75 = 0 (0.0%)<br>1 = 0 (0.0%) | 0 = 22 (88.0%)<br>0.25 = 3 (12.0%)<br>0.5 = 0 (0.0%)<br>0.75 = 0 (0.0%)<br>1 = 0 (0.0%) |
| | Handling small objects, like picking up a coin from a table? | 0 = No.<br>0.25 = Yes, a little difficult.<br>0.5 = Yes, somewhat difficult.<br>0.75 = Yes, very difficult.<br>1 = Unable to do so.<br>1 = Do not do on doctor's orders. | 0 = 41 (97.6%)<br>0.25 = 0 (0.0%)<br>0.5 = 0 (0.0%)<br>0.75 = 1 (2.4%)<br>1 = 0 (0.0%) | 0 = 39 (92.9%)<br>0.25 = 2 (4.8%)<br>0.5 = 0 (0.0%)<br>0.75 = 1 (2.4%)<br>1 = 0 (0.0%) | 0 = 24 (96.0%)<br>0.25 = 1 (4.0%)<br>0.5 = 0 (0.0%)<br>0.75 = 0 (0.0%)<br>1 = 0 (0.0%) | 0 = 23 (92.0%)<br>0.25 = 2 (8.0%)<br>0.5 = 0 (0.0%)<br>0.75 = 0 (0.0%)<br>1 = 0 (0.0%) |

| Question | Deficit | Coding | Frequency N (sample %) | | | |
|---|---|---|---|---|---|---|
| | | | Center-based CR Responses | | Virtual CR Responses | |
| | | | Admission | Follow-up | Admission | Follow-up |
| | Standing for a long period, around 15 minutes? | 0 = No.<br>0.25 = Yes, a little difficult.<br>0.5 = Yes, somewhat difficult.<br>0.75 = Yes, very difficult.<br>1 = Unable to do so.<br>1 = Do not do on doctor's orders. | 0 = 31 (73.8%)<br>0.25 = 7 (16.7%)<br>0.5 = 2 (4.8%)<br>0.75 = 2 (4.8%)<br>1 = 0 (0.0%) | 0 = 32 (76.2%)<br>0.25 = 4 (9.5%)<br>0.5 = 6 (14.3%)<br>0.75 = 0 (0.0%)<br>1 = 0 (0.0%) | 0 = 22 (88.0%)<br>0.25 = 0 (0.0%)<br>0.5 = 2 (8.0%)<br>0.75 = 1 (4.0%)<br>1 = 0 (0.0%) | 0 = 19 (76.0%)<br>0.25 = 5 (20.0%)<br>0.5 = 0 (0.0%)<br>0.75 = 1 (4.0%)<br>1 = 0 (0.0%) |
| | Standing up after sitting in a chair? | 0 = No.<br>0.25 = Yes, a little difficult.<br>0.5 = Yes, somewhat difficult.<br>0.75 = Yes, very difficult.<br>1 = Unable to do so.<br>1 = Do not do on doctor's orders. | 0 = 26 (61.9%)<br>0.25 = 14 (33.3%)<br>0.5 = 0 (0.0%)<br>0.75 = 2 (4.8%)<br>1 = 0 (0.0%) | 0 = 31 (73.8%)<br>0.25 = 9 (21.4%)<br>0.5 = 0 (0.0%)<br>0.75 = 2 (4.8%)<br>1 = 0 (0.0%) | 0 = 16 (64.0%)<br>0.25 = 9 (36.0%)<br>0.5 = 0 (0.0%)<br>0.75 = 0 (0.0%)<br>1 = 0 (0.0%) | 0 = 21 (84.0%)<br>0.25 = 3 (12.0%)<br>0.5 = 1 (4.0%)<br>0.75 = 0 (0.0%)<br>1 = 0 (0.0%) |
| | Walking alone up and down a flight of stairs? | 0 = No.<br>0.25 = Yes, a little difficult.<br>0.5 = Yes, somewhat difficult.<br>0.75 = Yes, very difficult.<br>1 = Unable to do so.<br>1 = Do not do on doctor's orders. | 0 = 34 (81.0%)<br>0.25 = 5 (11.9%)<br>0.5 = 0 (0.0%)<br>0.75 = 3 (7.1%)<br>1 = 0 (0.0%) | 0 = 32 (76.2%)<br>0.25 = 7 (16.7%)<br>0.5 = 3 (7.1%)<br>0.75 = 0 (0.0%)<br>1 = 0 (0.0%) | 0 = 20 (80.0%)<br>0.25 = 5 (20.0%)<br>0.5 = 0 (0.0%)<br>0.75 = 0 (0.0%)<br>1 = 0 (0.0%) | 0 = 21 (84.0%)<br>0.25 = 3 (12.0%)<br>0.5 = 1 (4.0%)<br>0.75 = 0 (0.0%)<br>1 = 0 (0.0%) |
| | Walking 2-3 neighborhood blocks? | 0 = No.<br>0.25 = Yes, a little difficult.<br>0.5 = Yes, somewhat difficult.<br>0.75 = Yes, very difficult.<br>1 = Unable to do so.<br>1 = Do not do on doctor's orders. | 0 = 32 (76.2%)<br>0.25 = 3 (7.1%)<br>0.5 = 3 (7.1%)<br>0.75 = 3 (7.1%)<br>1 = 1 (2.4%) | 0 = 35 (83.3%)<br>0.25 = 2 (4.8%)<br>0.5 = 3 (7.1%)<br>0.75 = 2 (4.8%)<br>1 = 0 (0.0%) | 0 = 18 (72.0%)<br>0.25 = 6 (24.0%)<br>0.5 = 1 (4.0%)<br>0.75 = 0 (0.0%)<br>1 = 0 (0.0%) | 0 = 19 (76.0%)<br>0.25 = 4 (16.0%)<br>0.5 = 1 (4.0%)<br>0.75 = 1 (4.0%)<br>1 = 0 (0.0%) |

| Question | Deficit | Coding | Frequency N (sample %) | | | |
|---|---|---|---|---|---|---|
| | | | Center-based CR Responses | | Virtual CR Responses | |
| | | | Admission | Follow-up | Admission | Follow-up |
| | Making a bed? | 0 = No.<br>0.25 = Yes, a little difficult.<br>0.5 = Yes, somewhat difficult.<br>0.75 = Yes, very difficult.<br>1 = Unable to do so.<br>1 = Do not do on doctor's orders. | 0 = 37 (88.1%)<br>0.25 = 3 (7.1%)<br>0.5 = 1 (2.4%)<br>0.75 = 1 (2.4%)<br>1 = 0 (0.0%) | 0 = 38 (90.5%)<br>0.25 = 1 (2.4%)<br>0.5 = 3 (7.1%)<br>0.75 = 0 (0.0%)<br>1 = 0 (0.0%) | 0 = 25 (100.0%)<br>0.25 = 0 (0.0%)<br>0.5 = 0 (0.0%)<br>0.75 = 0 (0.0%)<br>1 = 0 (0.0%) | 0 = 24 (96.0%)<br>0.25 = 1 (4.0%)<br>0.5 = 0 (0.0%)<br>0.75 = 0 (0.0%)<br>1 = 0 (0.0%) |
| | Washing your back? | 0 = No.<br>0.25 = Yes, a little difficult.<br>0.5 = Yes, somewhat difficult.<br>0.75 = Yes, very difficult.<br>1 = Unable to do so.<br>1 = Do not do on doctor's orders. | 0 = 33 (78.6%)<br>0.25 = 4 (9.5%)<br>0.5 = 3 (7.1%)<br>0.75 = 0 (0.0%)<br>1 = 2 (4.8%) | 0 = 37 (88.1%)<br>0.25 = 2 (4.8%)<br>0.5 = 2 (4.8%)<br>0.75 = 0 (0.0%)<br>1 = 1 (2.4%) | 0 = 17 (68.0%)<br>0.25 = 5 (20.0%)<br>0.5 = 1 (4.0%)<br>0.75 = 1 (4.0%)<br>1 = 1 (4.0%) | 0 = 16 (64.0%)<br>0.25 = 5 (20.0%)<br>0.5 = 1 (4.0%)<br>0.75 = 0 (0.0%)<br>1 = 3 (12.0%) |
| | Using a knife to cut food? | 0 = No.<br>0.25 = Yes, a little difficult.<br>0.5 = Yes, somewhat difficult.<br>0.75 = Yes, very difficult.<br>1 = Unable to do so.<br>1 = Do not do on doctor's orders. | 0 = 39 (92.9%)<br>0.25 = 2 (4.8%)<br>0.5 = 1 (2.4%)<br>0.75 = 0 (0.0%)<br>1 = 0 (0.0%) | 0 = 40 (95.2%)<br>0.25 = 1 (2.4%)<br>0.5 = 0 (0.0%)<br>0.75 = 1 (2.4%)<br>1 = 0 (0.0%) | 0 = 25 (100.0%)<br>0.25 = 0 (0.0%)<br>0.5 = 0 (0.0%)<br>0.75 = 0 (0.0%)<br>1 = 0 (0.0%) | 0 = 25 (100.0%)<br>0.25 = 0 (0.0%)<br>0.5 = 0 (0.0%)<br>0.75 = 0 (0.0%)<br>1 = 0 (0.0%) |
| | Recreational or work activities in which you take some force or impact through your arm, shoulder, or hand? | 0 = No.<br>0.25 = Yes, a little difficult.<br>0.5 = Yes, somewhat difficult.<br>0.75 = Yes, very difficult.<br>1 = Unable to do so.<br>1 = Do not do on doctor's orders. | 0 = 32 (76.2%)<br>0.25 = 6 (14.3%)<br>0.5 = 1 (2.4%)<br>0.75 = 2 (4.8%)<br>1 = 1 (2.4%) | 0 = 33 (78.6%)<br>0.25 = 6 (14.3%)<br>0.5 = 0 (0.0%)<br>0.75 = 2 (4.8%)<br>1 = 1 (2.4%) | 0 = 23 (92.0%)<br>0.25 = 0 (0.0%)<br>0.5 = 0 (0.0%)<br>0.75 = 0 (0.0%)<br>1 = 1 (4.0%)<br>Missing = 1 (4.0%) | 0 = 19 (76.0%)<br>0.25 = 4 (16.0%)<br>0.5 = 1 (4.0%)<br>0.75 = 0 (0.0%)<br>1 = 1 (4.0%) |

| Question | Deficit | Coding | Frequency N (sample %) | | | |
|---|---|---|---|---|---|---|
| | | | Center-based CR Responses | | Virtual CR Responses | |
| | | | Admission | Follow-up | Admission | Follow-up |
| 4) Please answer the following.. | In General, would you say your health is...? | 0 = Excellent.<br>0.25 = Very Good.<br>0.5 = Good.<br>0.75 = Fair.<br>1 = Poor. | 0 = 2 (4.8%)<br>0.25 = 8 (19.0%)<br>0.5 = 23 (54.8%)<br>0.75 = 8 (19.0%)<br>1 = 1 (2.4%) | 0 = 8 (19.0%)<br>0.25 = 10 (23.8%)<br>0.5 = 17 (40.5%)<br>0.75 = 6 (14.3%)<br>1 = 1 (2.4%) | 0 = 0 (0.0%)<br>0.25 = 3 (12.0%)<br>0.5 = 13 (52.0%)<br>0.75 = 8 (32.0%)<br>1 = 1 (4.0%) | 0 = 3 (12.0%)<br>0.25 = 3 (12.0%)<br>0.5 = 12 (48.0%)<br>0.75 = 7 (28.0%)<br>1 = 0 (0.0%) |
| | Is your eyesight, using glasses or corrective lens if you use them...? | 0 = Excellent.<br>0.25 = Very Good.<br>0.5 = Good.<br>0.75 = Fair.<br>1 = Poor. | 0 = 5 (11.9%)<br>0.25 = 15 (35.7%)<br>0.5 = 21 (50.0%)<br>0.75 = 1 (2.4%)<br>1 = 0 (0.0%) | 0 = 10 (23.8%)<br>0.25 = 16 (38.1%)<br>0.5 = 16 (38.1%)<br>0.75 = 0 (0.0%)<br>1 = 0 (0.0%) | 0 = 3 (12.0%)<br>0.25 = 9 (36.0%)<br>0.5 = 9 (36.0%)<br>0.75 = 2 (8.0%)<br>1 = 1 (4.0%)<br>Missing = 1 (4.0%) | 0 = 5 (20.0%)<br>0.25 = 6 (24.0%)<br>0.5 = 10 (40.0%)<br>0.75 = 2 (8.0%)<br>1 = 2 (8.0%) |
| | Is your hearing, using a hearing aid if you use one...? | 0 = Excellent.<br>0.25 = Very Good.<br>0.5 = Good.<br>0.75 = Fair.<br>1 = Poor. | 0 = 6 (14.3%)<br>0.25 = 15 (35.7%)<br>0.5 = 15 (35.7%)<br>0.75 = 6 (14.3%)<br>1 = 0 (0.0%) | 0 = 13 (31.0%)<br>0.25 = 14 (33.3%)<br>0.5 = 10 (23.8%)<br>0.75 = 4 (9.5%)<br>1 = 1 (2.4%) | 0 = 11 (44.0%)<br>0.25 = 0 (0.0%)<br>0.5 = 0 (0.0%)<br>0.75 = 0 (0.0%)<br>1 = 13 (52.0%)<br>Missing = 1 (4.0%) | 0 = 7 (28.0%)<br>0.25 = 3 (12.0%)<br>0.5 = 11 (44.0%)<br>0.75 = 4 (16.0%)<br>1 = 0 (0.0%) |
| 5) Do you consider yourself.. | Just about right.<br>Overweight?<br>Underweight? | 0 = Just about right.<br>1 = Overweight<br>1 = Underweight | 0 = 9 (21.4%)<br>1 = 33 (78.6%) | 0 = 13 (31.0%)<br>1 = 29 (69.0%) | 0 = 9 (36.0%)<br>1 = 16 (64.0%) | 0 = 10 (40.0%)<br>1 = 15 (60.0%) |

| Question | Deficit | Coding | Frequency N (sample %) | | | |
|---|---|---|---|---|---|---|
| | | | Center-based CR Responses | | Virtual CR Responses | |
| | | | Admission | Follow-up | Admission | Follow-up |
| 6) How many times have you.. | Had a fall in the past 12 months that was serious enough to limit some of your normal activities? For example, the fall resulted in a broken bone, bad cut, or sprain? | 0 = None.<br>1 = Once<br>2 = Twice or more | 0 = 37 (88.1%)<br>1 = 4 (9.5%)<br>2 = 1 (2.4%) | 0 = 40 (95.2%)<br>1 = 1 (2.4%)<br>2 = 1 (2.4%) | 0 = 23 (92.0%)<br>1 = 1 (4.0%)<br>2 = 1 (4.0%) | 0 = 23 (92.0%)<br>1 = 1 (4.0%)<br>2 = 1 (4.0%) |
| 7) In the past week how often did you feel.. | That everything you did was an effort? | 0 = Rarely or never (<1 day).<br>0.33 = Some of the time (1-2).<br>0.66 = Occasionally (3-4).<br>1 = All of the time (5-7 days). | 0 = 29 (69.0%)<br>0.33 = 7 (16.7%)<br>0.66 = 2 (4.8%)<br>1 = 4 (9.5%) | 0 = 28 (66.7%)<br>0.33 = 10 (23.8%)<br>0.66 = 2 (4.8%)<br>1 = 2 (4.8%) | 0 = 15 (60.0%)<br>0.33 = 7 (28.0%)<br>0.66 = 3 (12.0%)<br>1 = 0 (0.0%) | 0 = 16 (64.0%)<br>0.33 = 6 (24.0%)<br>0.66 = 3 (12.0%)<br>1 = 0 (0.0%) |
| | Lonely? | 0 = Rarely or never (<1 day).<br>0.33 = Some of the time (1-2).<br>0.66 = Occasionally (3-4).<br>1 = All of the time (5-7 days). | 0 = 35 (83.3%)<br>0.33 = 2 (4.8%)<br>0.66 = 3 (7.1%)<br>1 = 2 (4.8%) | 0 = 33 (78.6%)<br>0.33 = 5 (11.9%)<br>0.66 = 2 (4.8%)<br>1 = 2 (4.8%) | 0 = 18 (72.0%)<br>0.33 = 5 (20.0%)<br>0.66 = 2 (8.0%)<br>1 = 0 (0.0%) | 0 = 16 (64.0%)<br>0.33 = 3 (12.0%)<br>0.66 = 2 (8.0%)<br>1 = 2 (8.0%) |
| | That you could not “get going”? | 0 = Rarely or never (<1 day).<br>0.33 = Some of the time (1-2).<br>0.66 = Occasionally (3-4).<br>1 = All of the time (5-7 days). | 0 = 29 (69.0%)<br>0.33 = 9 (21.4%)<br>0.66 = 3 (7.1%)<br>1 = 1 (2.4%) | 0 = 29 (69.0%)<br>0.33 = 9 (21.4%)<br>0.66 = 2 (4.8%)<br>1 = 2 (4.8%) | 0 = 15 (60.0%)<br>0.33 = 8 (32.0%)<br>0.66 = 2 (8.0%)<br>1 = 0 (0.0%) | 0 = 16 (64.0%)<br>0.33 = 7 (28.0%)<br>0.66 = 1 (4.0%)<br>1 = 1 (4.0%) |

| Question | Deficit | Coding | Frequency N (sample %) | | | |
|---|---|---|---|---|---|---|
| | | | Center-based CR Responses | | Virtual CR Responses | |
| | | | Admission | Follow-up | Admission | Follow-up |
| 8) In the past 12 months, have you seen a doctor for.. | Pneumonia? | 0 = No<br>1 = Yes | 0 = 40 (95.2%)<br>1 = 2 (4.8%) | 0 = 40 (95.2%)<br>1 = 2 (4.8%) | 0 = 25 (100.0%)<br>1 = 0 (0.0%) | 0 = 25 (100.0%)<br>1 = 0 (0.0%) |
| | Urinary tract infection | 0 = No<br>1 = Yes | 0 = 39 (92.9%)<br>1 = 3 (7.1%) | 0 = 39 (92.9%)<br>1 = 1 (2.4%)<br>Missing = 1 (2.4%) | 0 = 23 (92.0%)<br>1 = 2 (8.0%) | 0 = 24 (96.0%)<br>1 = 1 (4.0%) |
| 9) Has a doctor ever told you that you.. | have osteoarthritis in the knee? | 0 = No<br>1 = Yes | 0 = 36 (85.7%)<br>1 = 6 (14.3%) | 0 = 36 (85.7%)<br>1 = 6 (14.3%) | 0 = 21 (84.0%)<br>1 = 4 (16.0%) | 0 = 21 (84.0%)<br>1 = 4 (16.0%) |
| | have osteoarthritis in the hip? | 0 = No<br>1 = Yes | 0 = 36 (85.7%)<br>1 = 6 (14.3%) | 0 = 36 (85.7%)<br>1 = 6 (14.3%) | 0 = 22 (88.0%)<br>1 = 3 (12.0%) | 0 = 22 (88.0%)<br>1 = 3 (12.0%) |
| | have osteoarthritis in one or both hands? | 0 = No<br>1 = Yes | 0 = 39 (92.9%)<br>1 = 3 (7.1%) | 0 = 39 (92.9%)<br>1 = 3 (7.1%) | 0 = 22 (88.0%)<br>1 = 3 (12.0%) | 0 = 22 (88.0%)<br>1 = 3 (12.0%) |
| | have rheumatoid arthritis? | 0 = No<br>1 = Yes | 0 = 40 (95.2%)<br>1 = 2 (4.8%) | 0 = 40 (95.2%)<br>1 = 2 (4.8%) | 0 = 25 (100.0%)<br>1 = 0 (0.0%) | 0 = 25 (100.0%)<br>1 = 0 (0.0%) |
| | have any other type of arthritis? | 0 = No<br>1 = Yes | 0 = 35 (83.3%)<br>1 = 7 (16.7%) | 0 = 35 (83.3%)<br>1 = 7 (16.7%) | 0 = 20 (80.0%)<br>1 = 5 (20.0%) | 0 = 20 (80.0%)<br>1 = 5 (20.0%) |

| Question | Deficit | Coding | Frequency N (sample %) | | | |
|---|---|---|---|---|---|---|
| | | | **Center-based CR Responses** | | **Virtual CR Responses** | |
| | | | Admission | Follow-up | Admission | Follow-up |
| | have/had any of the following- emphysema, chronic bronchitis, chronic obstructive pulmonary disease (COPD), or chronic changes in lungs due to smoking? | 0 = No<br>1 = Yes | 0 = 37 (88.1%)<br>1 = 5 (11.9%) | 0 = 37 (88.1%)<br>1 = 5 (11.9%) | 0 = 24 (96.0%)<br>1 = 1 (4.0%) | 0 = 24 (96.0%)<br>1 = 1 (4.0%) |
| | have high blood pressure or hypertension? | 0 = No<br>1 = Yes | 0 = 15 (35.7%)<br>1 = 27 (64.3%) | 0 = 15 (35.7%)<br>1 = 27 (64.3%) | 0 = 11 (44.0%)<br>1 = 14 (56.0%) | 0 = 11 (44.0%)<br>1 = 14 (56.0%) |
| | have diabetes, borderline diabetes or that your blood sugar is high? | 0 = No<br>1 = Yes | 0 = 34 (81.0%)<br>1 = 8 (19.0%) | 0 = 34 (81.0%)<br>1 = 8 (19.0%) | 0 = 19 (76.0%)<br>1 = 6 (24.0%) | 0 = 19 (76.0%)<br>1 = 6 (24.0%) |
| | have heart disease (including congestive heart failure or CHF)? | 0 = No<br>1 = Yes | 0 = 12 (28.6%)<br>1 = 30 (71.4%) | 0 = 12 (28.6%)<br>1 = 30 (71.4%) | 0 = 8 (32.0%)<br>1 = 17 (68.0%) | 0 = 8 (32.0%)<br>1 = 17 (68.0%) |
| | have angina (or chest pain due to heart disease)? | 0 = No<br>1 = Yes | 0 = 29 (69.0%)<br>1 = 13 (31.0%) | 0 = 29 (69.0%)<br>1 = 13 (31.0%) | 0 = 19 (76.0%)<br>1 = 6 (24.0%) | 0 = 19 (76.0%)<br>1 = 6 (24.0%) |

| Question | Deficit | Coding | Frequency N (sample %) | | | |
|---|---|---|---|---|---|---|
| | | | Center-based CR Responses | | Virtual CR Responses | |
| | | | Admission | Follow-up | Admission | Follow-up |
| | have had a heart attack, or myocardial infarction? | 0 = No<br>1 = Yes | 0 = 21 (50.0%)<br>1 = 21 (50.0%) | 0 = 21 (50.0%)<br>1 = 21 (50.0%) | 0 = 10 (40.0%)<br>1 = 15 (60.0%) | 0 = 10 (40.0%)<br>1 = 15 (60.0%) |
| | have peripheral vascular disease or poor circulation in your limbs? | 0 = No<br>1 = Yes | 0 = 41 (97.6%)<br>1 = 1 (2.4%) | 0 = 41 (97.6%)<br>1 = 1 (2.4%) | 0 = 25 (100.0%)<br>1 = 0 (0.0%) | 0 = 25 (100.0%)<br>1 = 0 (0.0%) |
| | have experienced a stroke or CVA (cerebrovascular accident)? | 0 = No<br>1 = Yes | 0 = 2 (4.8%)<br>1 = 40 (95.2%) | 0 = 2 (4.8%)<br>1 = 40 (95.2%) | 0 = 25 (100.0%)<br>1 = 0 (0.0%) | 0 = 25 (100.0%)<br>1 = 0 (0.0%) |
| | have experienced a mini-stroke or TIA (transient ischemic attack)? | 0 = No<br>1 = Yes | 0 = 40 (95.2%)<br>1 = 2 (4.8%) | 0 = 40 (95.2%)<br>1 = 2 (4.8%) | 0 = 25 (100.0%)<br>1 = 0 (0.0%) | 0 = 25 (100.0%)<br>1 = 0 (0.0%) |
| | have a memory problem? | 0 = No<br>1 = Yes | 0 = 42 (100.0%)<br>1 = 0 (0.0%) | 0 = 42 (100.0%)<br>1 = 0 (0.0%) | 0 = 25 (100.0%)<br>1 = 0 (0.0%) | 0 = 25 (100.0%)<br>1 = 0 (0.0%) |
| | have dementia or Alzheimer's disease? | 0 = No<br>1 = Yes | 0 = 42 (100.0%)<br>1 = 0 (0.0%) | 0 = 42 (100.0%)<br>1 = 0 (0.0%) | 0 = 25 (100.0%)<br>1 = 0 (0.0%) | 0 = 25 (100.0%)<br>1 = 0 (0.0%) |
| | had parkinsonism or Parkinson's disease? | 0 = No<br>1 = Yes | 0 = 42 (100.0%)<br>1 = 0 (0.0%) | 0 = 42 (100.0%)<br>1 = 0 (0.0%) | 0 = 25 (100.0%)<br>1 = 0 (0.0%) | 0 = 25 (100.0%)<br>1 = 0 (0.0%) |

| Question | Deficit | Coding | Frequency N (sample %) | | | |
|---|---|---|---|---|---|---|
| | | | Center-based CR Responses | | Virtual CR Responses | |
| | | | Admission | Follow-up | Admission | Follow-up |
| | have intestinal or stomach ulcers? | 0 = No<br>1 = Yes | 0 = 42 (100.0%)<br>1 = 0 (0.0%) | 0 = 42 (100.0%)<br>1 = 0 (0.0%) | 0 = 23 (92.0%)<br>1 = 2 (8.0%) | 0 = 23 (92.0%)<br>1 = 2 (8.0%) |
| | have a bowel disorder such as Crohn's disease, ulcerative colitis, or irritable bowel syndrome? | 0 = No<br>1 = Yes | 0 = 37 (88.1%)<br>1 = 5 (11.9%) | 0 = 37 (88.1%)<br>1 = 5 (11.9%) | 0 = 25 (100.0%)<br>1 = 0 (0.0%) | 0 = 25 (100.0%)<br>1 = 0 (0.0%) |
| | experience bowel incontinence? | 0 = No<br>1 = Yes | 0 = 42 (100.0%)<br>1 = 0 (0.0%) | 0 = 42 (100.0%)<br>1 = 0 (0.0%) | 0 = 25 (100.0%)<br>1 = 0 (0.0%) | 0 = 25 (100.0%)<br>1 = 0 (0.0%) |
| | experience urinary incontinence? | 0 = No<br>1 = Yes | 0 = 42 (100.0%)<br>1 = 0 (0.0%) | 0 = 42 (100.0%)<br>1 = 0 (0.0%) | 0 = 25 (100.0%)<br>1 = 0 (0.0%) | 0 = 25 (100.0%)<br>1 = 0 (0.0%) |
| | have cataracts? | 0 = No<br>1 = Yes | 0 = 41 (97.6%)<br>1 = 1 (2.4%) | 0 = 41 (97.6%)<br>1 = 1 (2.4%) | 0 = 25 (100.0%)<br>1 = 0 (0.0%) | 0 = 25 (100.0%)<br>1 = 0 (0.0%) |
| | have glaucoma? | 0 = No<br>1 = Yes | 0 = 42 (100.0%)<br>1 = 0 (0.0%) | 0 = 42 (100.0%)<br>1 = 0 (0.0%) | 0 = 24 (96.0%)<br>1 = 1 (4.0%) | 0 = 24 (96.0%)<br>1 = 1 (4.0%) |
| | have macular degeneration? | 0 = No<br>1 = Yes | 0 = 42 (100.0%)<br>1 = 0 (0.0%) | 0 = 42 (100.0%)<br>1 = 0 (0.0%) | 0 = 24 (96.0%)<br>1 = 1 (4.0%) | 0 = 24 (96.0%)<br>1 = 1 (4.0%) |

| Question | Deficit | Coding | Frequency N (sample %) | | | |
|---|---|---|---|---|---|---|
| | | | Center-based CR Responses | | Virtual CR Responses | |
| | | | Admission | Follow-up | Admission | Follow-up |
| | had cancer? | 0 = No<br>1 = Yes | 0 = 40 (95.2%)<br>1 = 2 (4.8%) | 0 = 40 (95.2%)<br>1 = 2 (4.8%) | 0 = 23 (92.0%)<br>1 = 2 (8.0%) | 0 = 23 (92.0%)<br>1 = 2 (8.0%) |
| | have osteoporosis, sometimes called low bone mineral density, or thin, brittle, or weak bones? | 0 = No<br>1 = Yes | 0 = 41 (97.6%)<br>1 = (2.4%) | 0 = 41 (97.6%)<br>1 = (2.4%) | 0 = 25 (100.0%)<br>1 = 0 (0.0%) | 0 = 25 (100.0%)<br>1 = 0 (0.0%) |
| | have back problems, excluding fibromyalgia and arthritis? | 0 = No<br>1 = Yes | 0 = 6 (14.3%)<br>1 = 36 (85.7%) | 0 = 6 (14.3%)<br>1 = 36 (85.7%) | 0 = 24 (96.0%)<br>1 = 1 (4.0%) | 0 = 24 (96.0%)<br>1 = 1 (4.0%) |
| | have an UNDER-active thyroid gland? | 0 = No<br>1 = Yes | 0 = 3 (7.1%)<br>1 = 39 (92.9%) | 0 = 3 (7.1%)<br>1 = 39 (92.9%) | 0 = 21 (84.0%)<br>1 = 4 (16.0%) | 0 = 21 (84.0%)<br>1 = 4 (16.0%) |
| | have an OVER-active thyroid gland? | 0 = No<br>1 = Yes | 0 = 42 (100.0%)<br>1 = 0 (0.0%) | 0 = 42 (100.0%)<br>1 = 0 (0.0%) | 0 = 25 (100.0%)<br>1 = 0 (0.0%) | 0 = 25 (100.0%)<br>1 = 0 (0.0%) |
| | have kidney disease or kidney failure? | 0 = No<br>1 = Yes | 0 = 5 (11.9%)<br>1 = 37 (88.1%) | 0 = 5 (11.9%)<br>1 = 37 (88.1%) | 0 = 24 (96.0%)<br>1 = 1 (4.0%) | 0 = 24 (96.0%)<br>1 = 1 (4.0%) |

Data are presented as n (%).
CLSA-FI Section 9: “Has a doctor ever told you that you..” used information that was collected by cardiac rehabilitation staff as part of routine care.

**Supplemental Table S3:** Cardiovascular biomarker variables added to the CLSA-FI

| **Variable** | **Cut-off Value for Deficit** |
|---|---|
| Systolic blood pressure | 0: 90-140 mmHg[a]<br>1: <90 or >140 mmHg |
| Diastolic blood pressure | 0: 60-90 mmHg<br>1: <60 or >90 mmHg |
| Resting heart rate | 0: 60-99 bpm[a]<br>1: <60 or >99 bpm |
| Total cholesterol | 0: <=6.2 mmol/L[a]<br>1: >6.2 mmol/L |
| High-density lipoprotein cholesterol | 0: >= 1.03 mmol/L<br>1: <1.03 mmol/L |
| Low-density lipoprotein cholesterol | 0: 0.98 -3.36 mmol/L<br>1: <0.98 or >3.36 mmol/L |
| Triglycerides | 0: <1.67 mmol/L<br>1: >=1.67 mmol/L |
| Creatinine | |
| - Women | 0: 45-90 umol/L[a]<br>1: <45 or >90 umol/L |
| - Men | 0: 60-110 umol/L<br>1:<60 or >110 umol/L |

[a]Abbreviations**:** BPM, beats per minute; umol/L, micromoles per litre; mmol/L, millimoles per litre; mmHg, millimoles of mercury. All CVD biomarker variables were routinely collected by cardiac rehabilitation staff.

**Supplemental Table S4:** Frailty changes by estimated marginal means with 95% CI for main and sensitivity analyses

| Outcome | Cardiac Rehabilitation Model | | P Value |
|---|---|---|---|
| | Center-based | Virtual | |
| **Main analysis:** | | | |
| Adjusted CLSA-FI[a] scores | | | |
| - **Admission** | **0.146 (0.140-0.151)** | **0.077 (0.071-0.084)** | ***.001**** |
| - **Follow-up** | **0.140 (0.120-0.162)** | **0.083 (0.059-0.107)** | ***.001**** |
| - Change between models (*F*(116,1) = 0.477) | | | .491 |
| **Sensitivity analysis:** | | | |
| Marginal estimates of FICVD[a] | | | |
| - **Admission** | **0.159 (0.154-0.164)** | **0.084 (0.078-0.091)** | ***.001**** |
| - **Follow-up** | **0.157 (0.138-0.177)** | **0.100 (0.077-0.123)** | ***.001**** |
| - Change between models (*F*(116,1) = 0.746) | | | .491 |
| Marginal estimates of listwise deletion | | | |
| - **Admission** | **0.148 (0.140-0.155)** | **0.064 (0.053-0.075)** | ***.001**** |
| - **Follow-up** | **0.132 (0.122-0.142)** | **0.070 (0.056-0.084)** | ***.001**** |
| - **Change between models (*F*(51,1) = 11.873)** | | | ***.001**** |

[a]Abbreviations: CLSA-FI, Canadian Longitudinal Study on Ageing Frailty Index; FICVD, frailty index including cardiovascular biomarkers. Data are presented as mean (95% CI). Computed using alpha = 0.05.

**Supplemental Table S5:** Simple slope analyses of frailty change by admission frailty[b] * CR[a] program model interaction on frailty change

| Simple Slope Analyses | R-square | Beta 95% CI[a] | | F-Statistic | P Value |
|---|---|---|---|---|---|
| | Beta | Lower | Upper | | |
| (Ref. is center-based CR[a]) | | | | | |
| **Main analysis** | **0.389** | **-0.813** | **-0.176** | ***4.709* (118, 16)** | ***.002**** |
| - **FI = 0.05** | **3.615** | **0.587** | **6.644** | | ***.018**** |
| - FI = 0.10 | 1.140 | -1.154 | 3.435 | | .322 |
| - FI = 0.15 | -1.334 | -3.870 | 1.200 | | .294 |
| - **FI = 0.20** | **-3.810** | **-7.369** | **-0.251** | | ***.034**** |
| - **FI = 0.25** | **-6.285** | **-11.181** | **-1.390** | | ***.011**** |
| **FICVD[a] sensitivity analysis** | **0.226** | **-0.906** | **-0.099** | ***2.105* (115, 16)** | ***.014**** |
| - FI = 0.05 | 2.662 | -1.451 | 6.775 | | .198 |
| - FI = 0.10 | 0.149 | -2.681 | 2.980 | | .915 |
| - FI = 0.15 | -2.362 | -5.055 | 0.329 | | .081 |
| - **FI = 0.20** | **-4.875** | **-8.699** | **-1.051** | | ***.012**** |
| - **FI = 0.25** | **-7.388** | **-12.878** | **-1.898** | | ***.008**** |
| Listwise deletion sensitivity analysis | 0.339 | -0.253 | 0.131 | *1.603* (50, 15) | .528 |
| - **FI = 0.05** | **1.648** | **0.059** | **3.237** | | ***.043**** |
| - **FI = 0.10** | **1.342** | **0.217** | **2.467** | | ***.020**** |
| - FI = 0.15 | 1.036 | -0.328 | 2.401 | | .135 |
| - FI = 0.20 | 0.730 | -1.347 | 2.808 | | .485 |
| - FI = 0.25 | 0.424 | -2.513 | 3.361 | | .773 |

[a]Abbreviations: CI, confidence interval; CR, cardiac rehabilitation; FICVD, frailty index including cardiovascular biomarkers. [b]Frailty values were multiplied by 100 to increase interpretability of findings, corresponding beta-coefficients relate to 1-unit increases in frailty. Computed using alpha = 0.05. Model used center-based CR as the reference.

**Supplemental Table S6**: Admission and follow-up mean differences in cardiovascular biomarkers by CR model

| **Cardiovascular Biomarker** | **Time** | **Cardiac Rehabilitation Model** | | **P Value** |
|---|---|---|---|---|
| | | Center-Based | Virtual | |
| Triglycerides | Admission | 1.76 ± 1.01 | 1.54 ± 0.76 | .168 |
| | Follow-up | 1.30 ± 0.09 | 1.04 ± 0.09 | .059 |
| Total cholesterol | Admission | 3.74 ± 1.07 | 3.43 ± 0.76 | .062 |
| | Follow-up | 3.19 ± 0.11 | 2.89 ± 0.09 | .051 |
| HDL-cholesterol[a] | Admission | 1.10 ± 0.28 | 1.13 ± 0.24 | .579 |
| | **Follow-up** | **1.26 ± 0.04** | **1.43 ± 0.05** | ***.010**** |
| LDL-cholesterol[a] | Admission | 1.85 ± 0.84 | 1.59 ± 0.65 | .053 |
| | Follow-up | 2.33 ± 0.12 | 2.69 ± 0.15 | .073 |
| Creatine kinase | Admission | 110.15 ± 64.48 | 115.10 ± 75.66 | .685 |
| | Follow-up | 217.82 ± 15.92 | 268.72 ± 22.53 | .091 |
| Creatinine | Admission | 86.65 ± 35.41 | 77.28 ± 15.32 | .062 |
| | Follow-up | 124.20 ± 15.95 | 176.33 ± 30.61 | .111 |
| C-Reactive protein | Admission | 6.70 ± 16.57 | 4.03 ± 5.25 | .240 |
| | Follow-up | 5.43 ± 2.34 | 7.64 ± 4.16 | .628 |
| Systolic blood pressure | Admission | 122.72 ± 19.84 | 125.53 ± 15.02 | .371 |
| | Follow-up | 122.15 ± 2.05 | 121.17 ± 2.56 | .764 |
| Diastolic blood pressure | Admission | 72.19 ± 10.11 | 71.64 ± 9.05 | .746 |
| | Follow-up | 69.55 ± 1.28 | 71.02 ± 1.50 | .460 |
| Resting pulse | Admission | 66.89 ± 10.82 | 65.45 ± 10.73 | .447 |
| | Follow-up | 65.42 ± 1.63 | 69.05 ± 2.24 | .182 |

[a]Abbreviations: HDL, high-density lipoprotein; LDL, low-density lipoprotein. Data are presented as mean ± SD. Computed at alpha = 0.05.

**Supplemental Table S7:** Multivariable linear regression analysis of admission CLSA-FI[a,b] on change in cardiovascular biomarkers

| Cardiovascular Biomarker | Beta | Beta 95% CI[a] | | P Value |
|---|---|---|---|---|
| | | Lower | Upper | |
| - Triglycerides | -0.009 | -0.034 | 0.016 | .476 |
| - Total cholesterol | 0.004 | -0.023 | 0.032 | .725 |
| - High-density lipoprotein cholesterol | 0.001 | -0.008 | 0.011 | .753 |
| - Low-density lipoprotein cholesterol | -0.003 | -0.035 | 0.028 | .828 |
| - Creatine kinase | 1.806 | -3.435 | 7.047 | .492 |
| - Creatinine | -2.572 | -7.711 | 2.567 | .318 |
| - C-reactive protein | 0.085 | -0.636 | 0.807 | .813 |
| - Systolic blood pressure | -0.045 | -0.558 | 0.468 | .861 |
| - Diastolic blood pressure | 0.293 | -0.003 | 0.591 | .051 |
| - Resting pulse | 0.191 | -0.336 | 0.719 | .469 |

[a]Abbreviations: CI, confidence interval; CLSA-FI, Canadian Longitudinal Study on Ageing Frailty Index.
[b]CLSA-FI values were multiplied by 100 to increase interpretability of findings, corresponding beta-coefficients relate to 1-unit increases in CLSA-FI. Computed using alpha = 0.05.

**Supplemental Table S8:** Sensitivity analysis – linear regression of admission FICVD[a,b] on change in cardiovascular biomarkers

| **Cardiovascular Biomarker** | **Beta** | **Beta 95% CI**[a] | | **P Value** |
|---|---|---|---|---|
| | | Lower | Upper | |
| - Triglycerides | -0.008 | -0.036 | 0.019 | .563 |
| - Total cholesterol | 0.005 | -0.025 | 0.036 | .739 |
| - High-density lipoprotein cholesterol | 0.003 | -0.007 | 0.014 | .506 |
| - Low-density lipoprotein cholesterol | -0.004 | -0.039 | 0.031 | .814 |
| - Creatine kinase | 0.923 | -4.983 | 6.829 | .755 |
| - Creatinine | -3.162 | -8.926 | 2.600 | .274 |
| - C-reactive protein | 0.332 | -0.478 | 1.144 | .414 |
| - Systolic blood pressure | -0.069 | -0.636 | 0.497 | .805 |
| - **Diastolic blood pressure** | **0.336** | **0.008** | **0.664** | ***.042**** |
| - Resting pulse | 0.177 | -0.412 | 0.766 | .549 |

[a]Abbreviation: CI, confidence interval; FICVD, frailty index including cardiovascular biomarkers. [b]FICVD values were multiplied by 100 to increase interpretability of findings, corresponding beta-coefficients relate to 1-unit increases in FICVD. Computed using alpha = 0.05

**Supplemental Table S9:** Simple slope sensitivity analyses of cardiovascular biomarker change by admission FICVD[a,b] * CR[a] program model interaction

| **Cardiovascular Biomarker** | **R Square** - Beta | **Beta 95% CI[a]** Lower | Upper | **F-Statistic** | **P Value** |
|---|---|---|---|---|---|
| Simple slope analysis (Ref. is center-based CR[a]) | | | | | |
| **Triglycerides** | **0.134** | **-0.111** | **-0.003** | ***1.198* (115, 16)** | ***.035**** |
| - FI = 0.05 | 0.312 | -0.226 | 0.851 | | .248 |
| - FI = 0.10 | 0.024 | -0.348 | 0.397 | | .895 |
| - FI = 0.15 | -0.263 | -0.630 | 0.103 | | .153 |
| - **FI = 0.20** | **-0.551** | **-1.077** | **-0.024** | | ***.038**** |
| - **FI = 0.25** | **-0.839** | **-1.592** | **-0.086** | | ***.027**** |
| **Total cholesterol** | **0.233** | **-0.116** | **-0.000** | ***2.349* (115, 16)** | ***.048**** |
| - FI = 0.05 | 0.384 | -0.218 | 0.988 | | .204 |
| - FI = 0.10 | 0.094 | -0.320 | 0.509 | | .648 |
| - FI = 0.15 | -0.195 | -0.582 | 0.191 | | .314 |
| - FI = 0.20 | -0.485 | -1.030 | 0.059 | | .077 |
| - FI = 0.25 | -0.775 | -1.559 | 0.007 | | .050 |
| HDL-cholesterol[a] | 0.393 | -0.013 | 0.029 | *5.024* (115, 16) | .462 |
| - FI = 0.05 | -0.158 | -0.372 | 0.054 | | .140 |
| - FI = 0.10 | -0.118 | -0.267 | 0.029 | | .112 |
| - FI = 0.15 | -0.078 | -0.227 | 0.069 | | .290 |
| - FI = 0.20 | -0.038 | -0.251 | 0.174 | | .716 |
| - FI = 0.25 | 0.001 | -0.302 | 0.304 | | .994 |
| LDL-cholesterol[a] | 0.178 | -0.059 | 0.073 | *1.678* (115, 16) | .827 |
| - FI = 0.05 | -0.390 | -1.072 | 0.291 | | .253 |
| - FI = 0.10 | -0.354 | -0.829 | 0.120 | | .138 |
| - FI = 0.15 | -0.317 | -0.774 | 0.138 | | .166 |
| - FI = 0.20 | -0.281 | -0.924 | 0.361 | | .383 |
| - FI = 0.25 | -0.244 | -1.162 | 0.672 | | .594 |
| Creatine kinase | 0.127 | -18.865 | 3.319 | *1.128* (115, 16) | .164 |
| - FI = 0.05 | 30.044 | -82.273 | 142.361 | | .593 |
| - FI = 0.10 | -8.821 | -86.228 | 68.585 | | .820 |
| - FI = 0.15 | -47.687 | -121.988 | 26.614 | | .201 |
| - FI = 0.20 | -86.552 | -192.390 | 19.284 | | .104 |
| - FI = 0.25 | -125.418 | -277.189 | 26.352 | | .101 |

| Cardiovascular Biomarker | R Square<br>- Beta | Beta 95% CI[a] | | F-Statistic | P Value |
|---|---|---|---|---|---|
| | | Lower | Upper | | |
| Creatinine | 0.058 | -13.907 | 7.788 | *0.483* (115, 16) | .574 |
| - FI = 0.05 | 35.914 | -74.097 | 145.926 | | .515 |
| - FI = 0.10 | 20.617 | -55.741 | 96.975 | | .590 |
| - FI = 0.15 | 5.319 | -68.450 | 79.089 | | .885 |
| - FI = 0.20 | -9.978 | -114.558 | 94.601 | | .848 |
| - FI = 0.25 | -25.276 | -174.659 | 124.106 | | .735 |
| C-reactive protein | 0.260 | -1.135 | 1.948 | *2.717* (115, 16) | .599 |
| - FI = 0.05 | -6.269 | -21.973 | 9.433 | | .426 |
| - FI = 0.10 | -4.237 | -15.067 | 6.593 | | .435 |
| - FI = 0.15 | -2.205 | -12.543 | 8.133 | | .670 |
| - FI = 0.20 | -0.172 | -14.847 | 14.502 | | .981 |
| - FI = 0.25 | 1.859 | -19.180 | 22.900 | | .859 |
| Systolic blood pressure | 0.186 | -0.769 | 1.390 | *1.767* (115, 16) | .566 |
| - FI = 0.05 | -7.037 | -17.840 | 3.765 | | .195 |
| - FI = 0.10 | -5.485 | -12.984 | 2.012 | | .146 |
| - FI = 0.15 | -3.933 | -11.287 | 3.419 | | .286 |
| - FI = 0.20 | -2.381 | -12.881 | 8.118 | | .650 |
| - FI = 0.25 | -0.829 | -15.821 | 14.161 | | .912 |
| Diastolic blood pressure | 0.222 | -0.924 | 0.339 | *2.214* (115, 16) | .357 |
| - FI = 0.05 | 3.070 | -3.455 | 9.596 | | .348 |
| - FI = 0.10 | 1.609 | -2.888 | 6.106 | | .475 |
| - FI = 0.15 | 0.147 | -4.078 | 4.373 | | .944 |
| - FI = 0.20 | -1.314 | -7.271 | 4.642 | | .659 |
| - FI = 0.25 | -2.775 | -11.325 | 5.774 | | .517 |
| Resting pulse | 0.150 | -1.826 | 0.431 | *1.367* (115, 16) | .219 |
| - FI = 0.05 | 0.892 | -10.608 | 12.393 | | .876 |
| - FI = 0.10 | -2.594 | -10.581 | 5.392 | | .517 |
| - FI = 0.15 | -6.082 | -13.766 | 1.601 | | .116 |
| - FI = 0.20 | -9.569 | -20.434 | 1.294 | | .080 |
| - FI = 0.25 | -13.057 | -28.573 | 2.459 | | .095 |

[a]Abbreviations: CI, confidence interval; CR, cardiac rehabilitation; FICVD, frailty index including cardiovascular biomarkers; HDL, high-density lipoprotein; LDL, low-density lipoprotein. [b]FICVD values were multiplied by 100 to increase interpretability of findings, corresponding beta-coefficients relate to 1-unit increases in FICVD. Computed using alpha = 0.05

**Supplemental Table S10:** Listwise deletion sensitivity analysis - linear regression of admission CLSA-FI[a,b] on change in cardiovascular biomarkers

| **Cardiovascular Biomarker** | **Beta** | **Beta 95% CI[a]** | | **P Value** |
|---|---|---|---|---|
| | | Lower | Upper | |
| - Triglycerides | -0.012 | -0.042 | 0.018 | .429 |
| - Total cholesterol | 0.008 | -0.025 | 0.042 | .614 |
| - High-density lipoprotein cholesterol | 0.008 | -0.007 | 0.024 | .306 |
| - **Low-density lipoprotein cholesterol** | **0.051** | **0.004** | **0.098** | ***.033**** |
| - Creatine kinase | 3.678 | -2.807 | 10.164 | .266 |
| - Creatinine | 3.027 | -3.811 | 9.867 | .261 |
| - C-reactive protein | -0.306 | -1.281 | 0.668 | .532 |
| - Systolic blood pressure | -0.053 | -0.763 | 0.655 | .879 |
| - Diastolic blood pressure | 0.018 | -0.462 | 0.499 | .939 |
| - Resting pulse | 0.048 | -0.580 | 0.677 | .878 |

[a]Abbreviations: CI, confidence interval; CLSA-FI, Canadian Longitudinal Study on Ageing Frailty Index.
[b]CLSA-FI values were multiplied by 100 to increase interpretability of findings, corresponding beta-coefficients relate to 1-unit increases in CLSA-FI. Computed using alpha = 0.05.

**Supplemental Table S11:** Listwise deletion simple slope sensitivity analyses of frailty change by admission CLSA-FI[a,b] * CR[a] program model interaction

| Cardiovascular Biomarker | R Square | - Beta | Beta 95% CI[a] | | F-Statistic | P Value |
|---|---|---|---|---|---|---|
| | | | Lower | Upper | | |
| Simple slope analysis (Ref. is center-based CR) | | | | | | |
| Triglycerides | 0.608 | | -0.090 | 0.046 | *5.280* (51, 15) | .531 |
| - FI = 0.05 | | -0.075 | -0.643 | 0.493 | | .792 |
| - FI = 0.10 | | -0.183 | -0.585 | 0.219 | | .367 |
| - FI = 0.15 | | -0.291 | -0.776 | 0.194 | | .236 |
| - FI = 0.20 | | -0.398 | -1.137 | 0.339 | | .284 |
| - FI = 0.25 | | -0.506 | -1.550 | 0.536 | | .335 |
| Total cholesterol | 0.569 | | -0.120 | 0.029 | *4.495* (51, 15) | .229 |
| - FI = 0.05 | | 0.063 | -0.562 | 0.690 | | .838 |
| - FI = 0.10 | | -0.163 | -0.608 | 0.282 | | .466 |
| - FI = 0.15 | | -0.390 | -0.923 | 0.142 | | .148 |
| - FI = 0.20 | | -0.617 | -1.422 | 0.187 | | .130 |
| - FI = 0.25 | | -0.845 | -1.981 | 0.291 | | .143 |
| **HDL-cholesterol[a]** | **0.338** | | **0.001** | **0.069** | ***1.742* (51, 15)** | ***.040**** |
| - FI = 0.05 | | 0.008 | -0.276 | 0.292 | | .954 |
| - FI = 0.10 | | 0.187 | -0.014 | 0.388 | | .069 |
| - **FI = 0.15** | | **0.366** | **0.124** | **0.607** | | ***.003**** |
| - **FI = 0.20** | | **0.545** | **0.179** | **0.910** | | ***.004**** |
| - **FI = 0.25** | | **0.724** | **0.208** | **1.240** | | ***.007**** |
| **LDL-cholesterol[a]** | **0.475** | | **0.031** | **0.226** | ***3.077* (51, 15)** | ***.011**** |
| - FI = 0.05 | | -0.087 | -0.907 | 0.733 | | .832 |
| - FI = 0.10 | | 0.557 | -0.022 | 1.136 | | .060 |
| - **FI = 0.15** | | **1.201** | **0.512** | **1.890** | | ***.001**** |
| - **FI = 0.20** | | **1.845** | **0.801** | **2.889** | | ***<.001**** |
| - **FI = 0.25** | | **2.489** | **1.012** | **3.967** | | ***.001**** |
| **Creatine kinase** | **0.428** | | **2.633** | **30.136** | ***2.545* (51, 15)** | ***.020**** |
| - FI = 0.05 | | -20.311 | -133.550 | 92.927 | | .721 |
| - FI = 0.10 | | 61.612 | -17.591 | 140.816 | | .125 |
| - **FI = 0.15** | | **143.536** | **47.732** | **239.340** | | ***.004**** |
| - **FI = 0.20** | | **225.460** | **78.700** | **372.220** | | ***.003**** |
| - **FI = 0.25** | | **307.384** | **99.169** | **515.598** | | ***.004**** |

| **Cardiovascular Biomarker** | **R Square - Beta** | **Beta 95% CI[a]** | | **F-Statistic** | **P Value** |
|---|---|---|---|---|---|
| | | Lower | Upper | | |
| Creatinine | 0.138 | -6.794 | 23.793 | *0.445* (51, 15) | .272 |
| - FI = 0.05 | -19.892 | -145.771 | 105.986 | | .753 |
| - FI = 0.10 | 22.606 | -66.331 | 111.543 | | .613 |
| - FI = 0.15 | 65.104 | -42.923 | 173.132 | | .233 |
| - FI = 0.20 | 107.602 | -57.096 | 272.301 | | .197 |
| - FI = 0.25 | 150.100 | -82.873 | 383.075 | | .203 |
| C-reactive protein | 0.165 | -3.181 | 1.181 | *0.676* (51, 15) | .364 |
| - FI = 0.05 | 12.470 | -5.593 | 30.534 | | .173 |
| - FI = 0.10 | 7.469 | -5.365 | 20.304 | | .249 |
| - FI = 0.15 | 2.468 | -13.061 | 17.998 | | .751 |
| - FI = 0.20 | -2.532 | -26.104 | 21.039 | | .830 |
| - FI = 0.25 | -7.533 | -40.821 | 25.754 | | .652 |
| Systolic blood pressure | 0.412 | -1.962 | 1.269 | *2.384* (51, 15) | .669 |
| - FI = 0.05 | -9.590 | -23.091 | 3.909 | | .161 |
| - **FI = 0.10** | **-11.323** | **-20.856** | **-1.789** | | ***.021**** |
| - **FI = 0.15** | **-13.055** | **-24.462** | **-1.648** | | ***.026**** |
| - FI = 0.20 | -14.787 | -32.106 | 2.530 | | .093 |
| - FI = 0.25 | -16.520 | -41.022 | 7.530 | | .183 |
| **Diastolic blood pressure** | **0.473** | **0.358** | **2.339** | ***3.063* (51, 15)** | ***.008**** |
| - FI = 0.05 | -4.706 | -13.095 | 3.682 | | .267 |
| - FI = 0.10 | 2.039 | -3.927 | 8.006 | | .497 |
| - **FI = 0.15** | **8.785** | **1.720** | **15.850** | | ***.016**** |
| - **FI = 0.20** | **15.531** | **4.886** | **26.176** | | ***.005**** |
| - **FI = 0.25** | **22.277** | **7.250** | **37.303** | | ***.004**** |
| Resting pulse | 0.201 | -2.421 | 0.562 | *0.867* (51, 15) | .218 |
| - FI = 0.05 | 7.300 | -5.066 | 19.668 | | .243 |
| - FI = 0.10 | 2.654 | -6.076 | 11.384 | | .545 |
| - FI = 0.15 | -1.992 | -12.516 | 8.531 | | .706 |
| - FI = 0.20 | -6.638 | -22.655 | 9.378 | | .411 |
| - FI = 0.25 | -11.284 | -33.947 | 11.377 | | .323 |

[a]Abbreviations: CI, confidence interval; CLSA-FI, Canadian Longitudinal Study on Ageing Frailty Index; CR, cardiac rehabilitation; HDL, high-density lipoprotein; LDL, low-density lipoprotein. [b]CLSA-FI values were multiplied by 100 to increase interpretability of findings, corresponding beta-coefficients relate to 1-unit increases in CLSA-FI. Computed using alpha = 0.05.

## Supplemental Figures Package

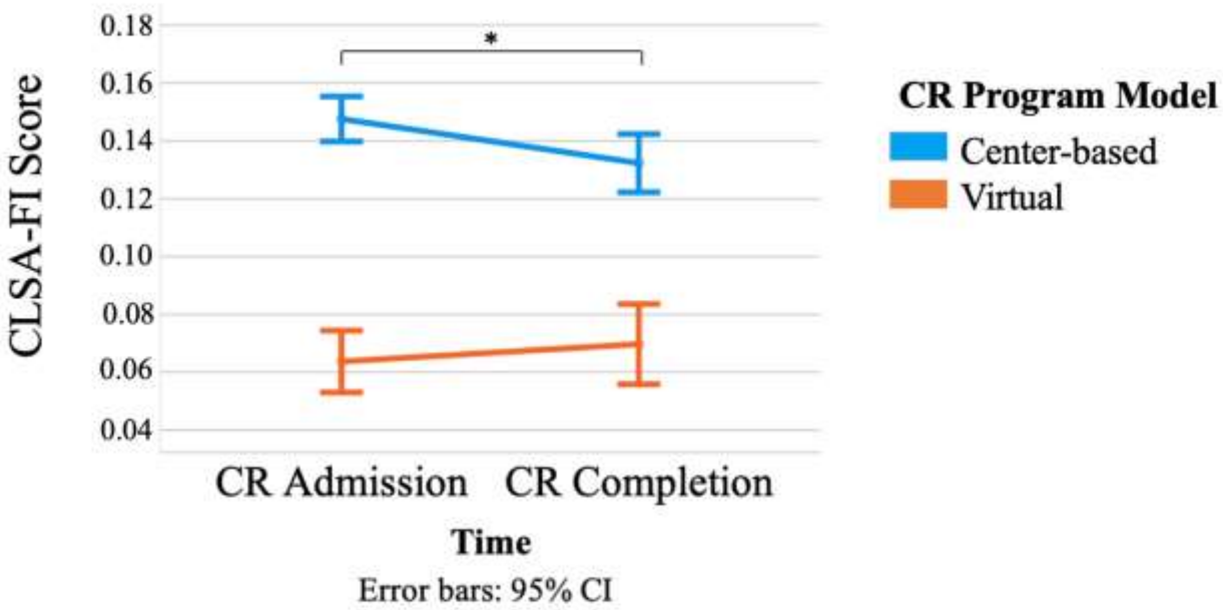


**Supplemental Figure S1.** Estimated marginal means of our listwise deletion sensitivity analysis on CLSA-FI scores at admission and follow-up (*p <.05).

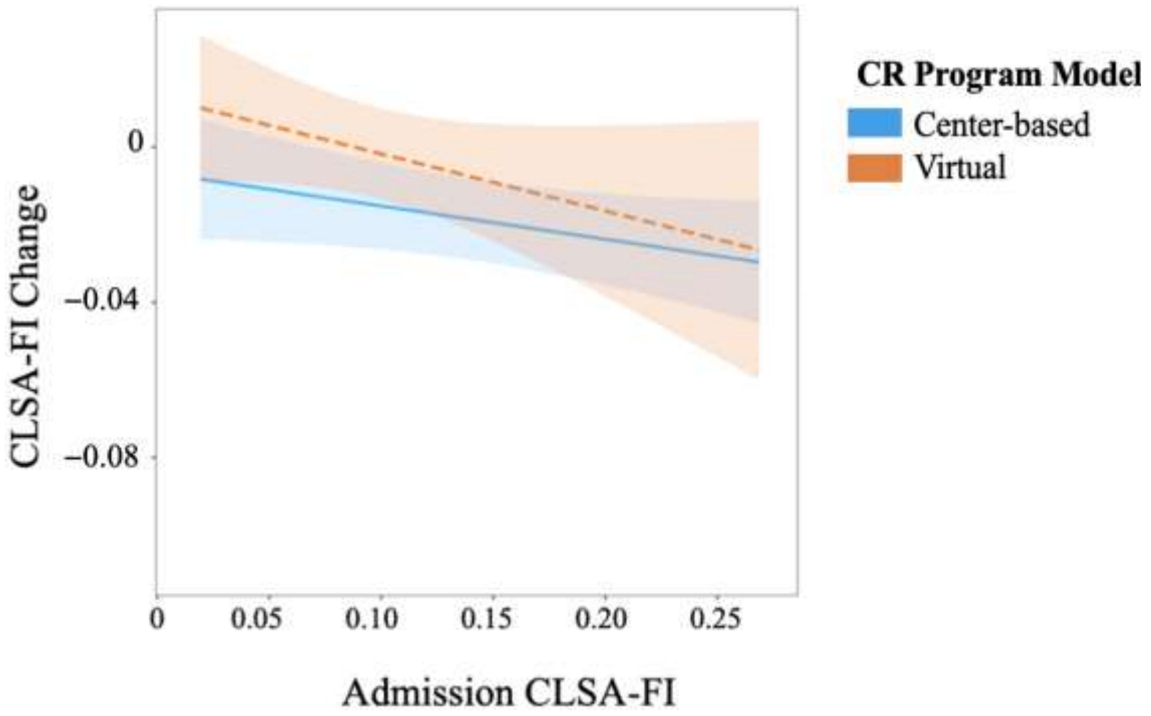


**Supplemental Figure S2.** Simple slope of listwise deletion sensitivity analysis predicting frailty change by admission frailty, stratified by CR model.

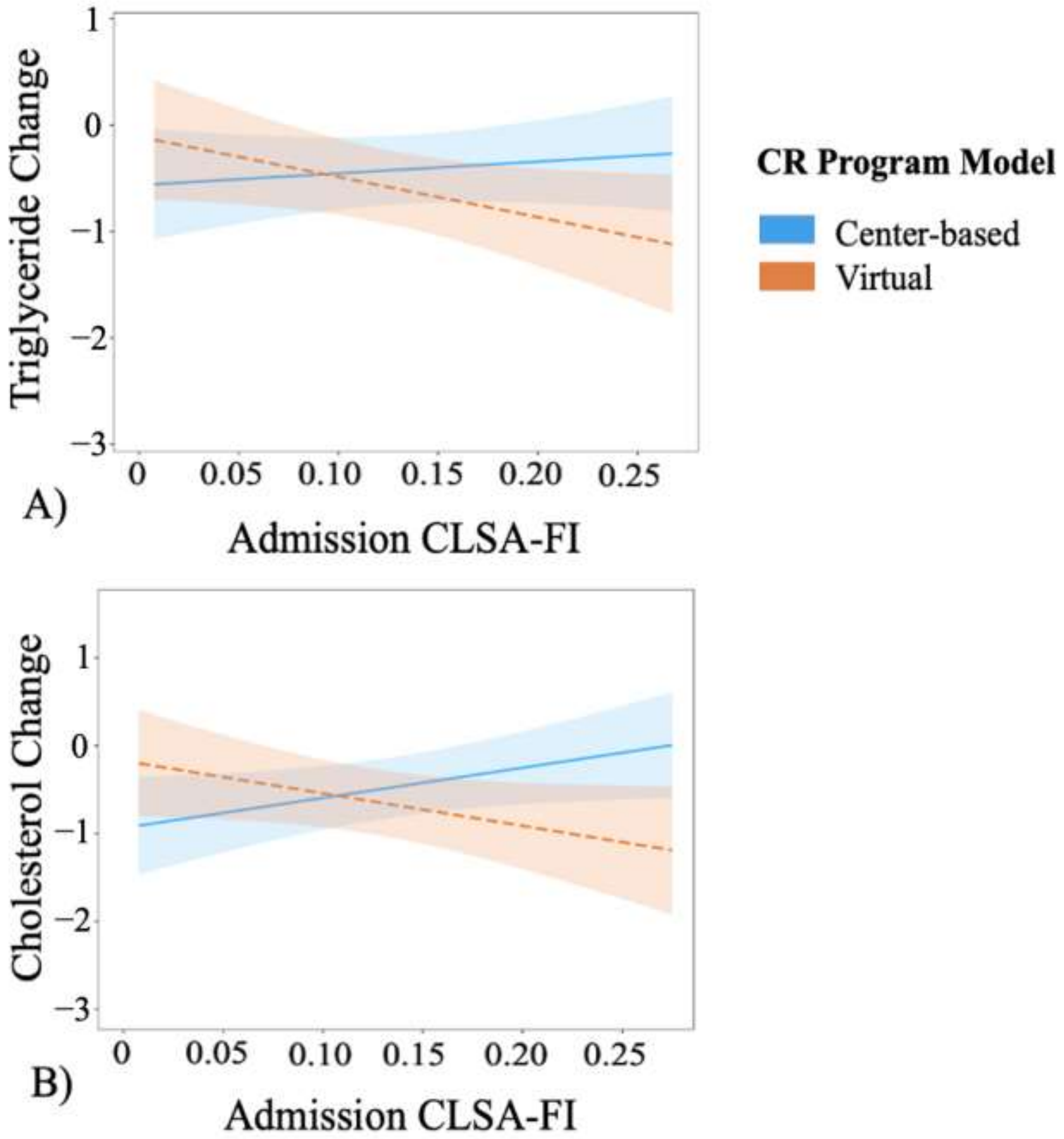


**Supplemental Figure S3.** Simple slopes of main analysis predicting: (**A)** Triglyceride change by admission frailty, stratified by CR model**, (B)** Cholesterol change by admission frailty, stratified by CR model.

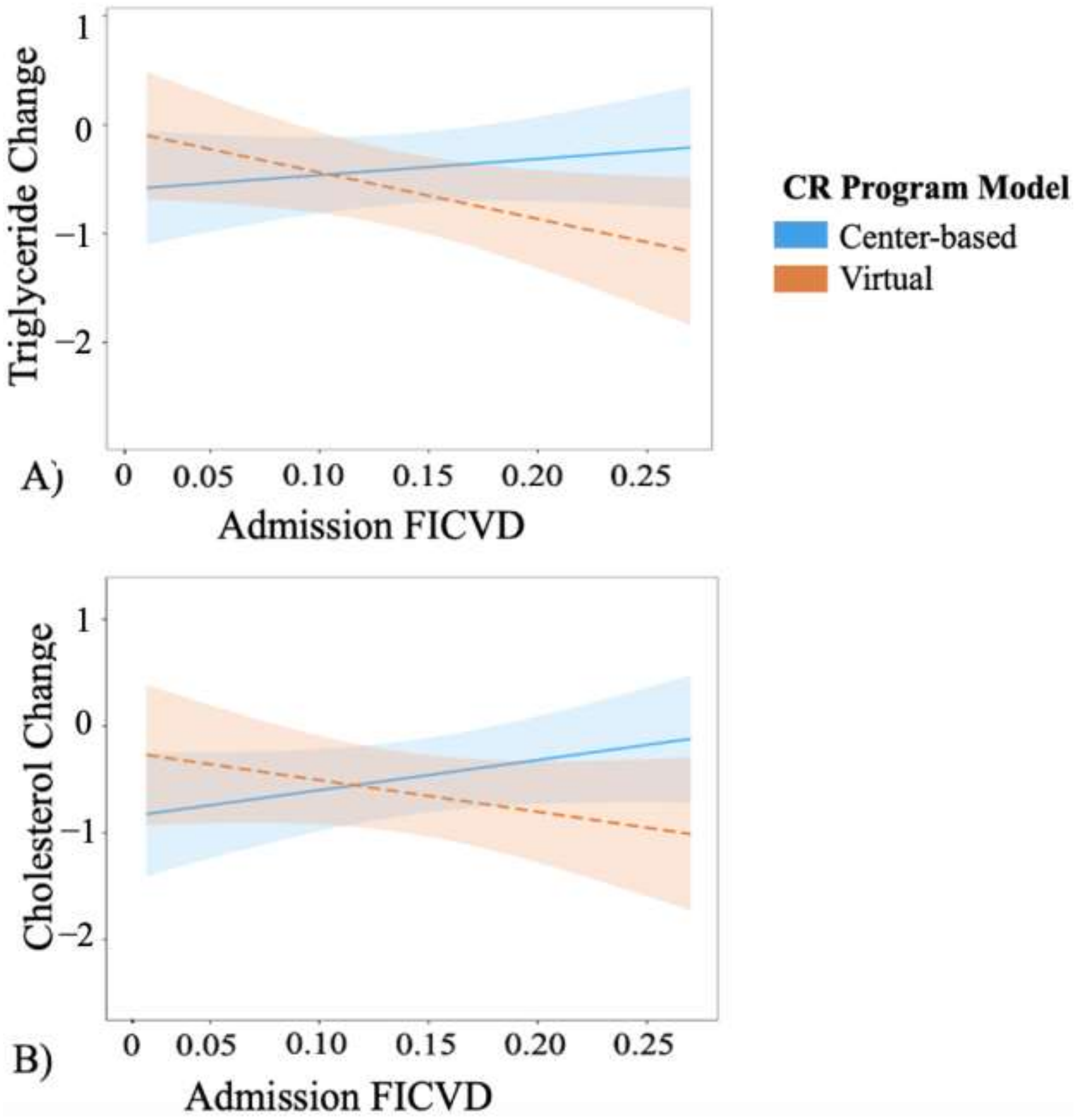


**Supplemental Figure S4.** Simple slopes of sensitivity analysis predicting: (**A)** Triglyceride change by admission FICVD score, stratified by CR model, **(B)** Cholesterol change by admission FICVD score, stratified by CR model.

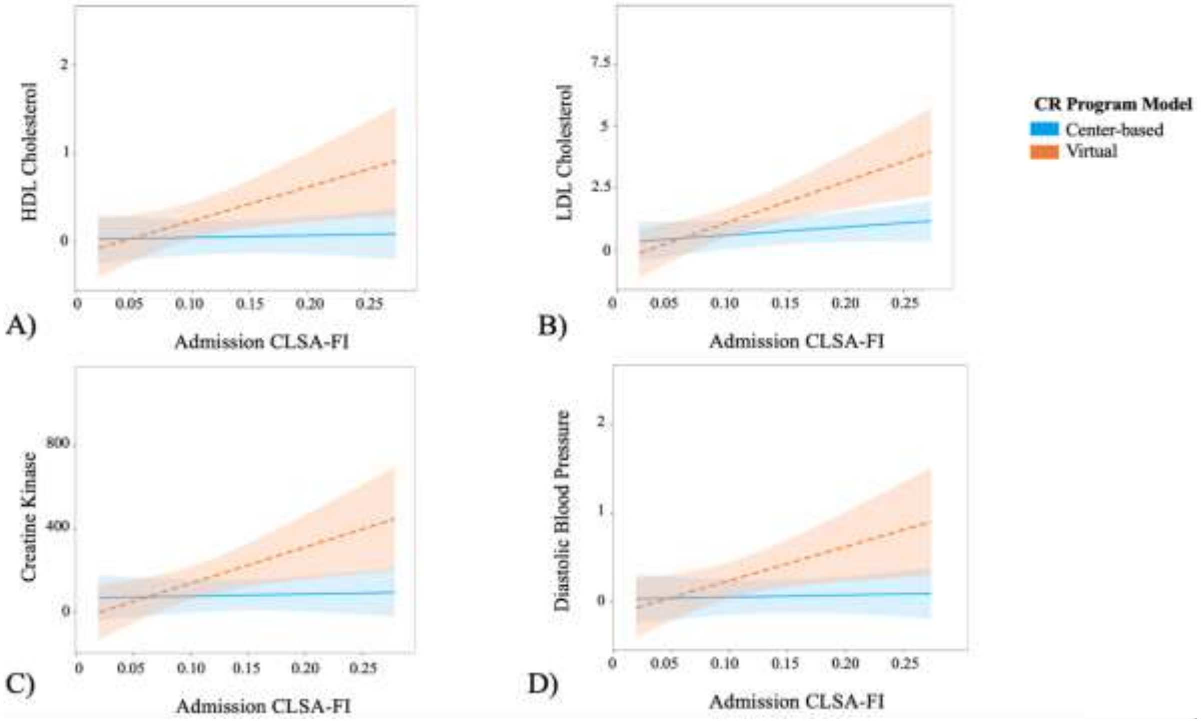


**Supplemental Figure S5.** Simple slopes of listwise deletion sensitivity analysis predicting (**A)** HDL cholesterol change by admission frailty, stratified by CR model, **(B)** LDL cholesterol change by admission frailty, stratified by CR model, (**C)** Creatine kinase change by admission frailty, stratified by CR model, **(D)** Diastolic blood pressure change by admission frailty, stratified by CR model.
